# Evaluation of Iron-induced Oxidative Stress and its Amelioration by Certain Herbs in Broilers

**DOI:** 10.4103/0971-6580.75861

**Published:** 2011

**Authors:** V. Ramakrishnan, A. Gopala Reddy, A. Rajasekher Reddy, C. Haritha

**Affiliations:** Department of Pharmacology and Toxicology, College of Veterinary Science, Rajendranagar, Hyderabad - 500 030, Andhra Pradesh, India; 1 Associate Dean, College of Veterinary Science, Korutla, Andhra Pradesh, India

**Keywords:** Broilers, herbs, iron, oxidative stress

## Abstract

A total of 225 male broiler chicks (Cobb strain) of day-old age were randomly divided into 15 groups consisting of 15 chicks in each group. Group 1 was maintained as basal diet control and group 2 on ferrous sulfate at 0.5% in feed throughout 6 wk as iron toxic control without any treatment. Groups 3-15 were maintained on FeSO_4_ at 0.5% in feed for the 4 wk (28 days) of study and thereafter administered with different herbs and their combinations for the remaining 2 wk. The blood samples were drawn from wing vein at the end of 4^th^ and 6^th^ weeks from the birds in each group for the assay of superoxide dismutase (SOD) and catalase. Sera samples were separated from the blood for the estimation of alanine transaminase (ALT) and serum creatinine. The birds were sacrificed at the end of 6^th^ wk and tissues were collected for the assay of reduced glutathione (GSH) and thiobarbituric acid reactive substances (TBARS) in liver and kidney homogenates. The activities of SOD, catalase and ALT, and the concentration of TBARS and serum creatinine were increased significantly (*P*<0.05), while the concentration of tissue GSH was decreased significantly (*P*<0.05) in all the groups as compared to basal diet control and the values showed significant improvement in groups 3-15 that were treated during the last 2 weeks. It is concluded that iron induces toxicity by generating reactive oxygen species, and antioxidant herbs are useful in treating the iron-induced toxicity.

## INTRODUCTION

Iron, by virtue of its ability to participate directly as a donor or acceptor in electron transfer reactions, is an essential trace element for cell function. This property makes iron the most common cofactor within the oxygen handling biological machinery.[1] However, the very property that enables iron to participate in oxygen metabolism explains its potential damaging effects: if not handled properly by the cell, iron interacts with molecular oxygen, generating reactive oxygen species (ROS) through Haber-Weiss and Fenton reactions.[2] Uncontrolled ROS production leads to oxidative damage of cellular components, a condition termed oxidative stress.[3] To prevent peroxidative tissue damage, there are protective mechanisms *in vivo*, such as an enzymatic defense system (antioxidant enzymes) and free-radical scavengers (antioxidants). Iron interferes with the activities of different antioxidants and hence may contribute to progression of liver damage and damage to other organ systems.[4]

Oxidative damage by free radicals can be prevented by the use of antioxidants such as ascorbic acid, tocopherols, carotenoids and certain herbs.[5] Of late, several herbs have been reported to counter the peroxidative stress due to several stressors. Keeping the above facts in view, an experimental study was planned to evaluate the mechanism of iron-induced oxidative stress and injury to the biological system, and to evaluate the therapeutic potential of certain herbs and herbal combinations against experimental iron toxicosis in broilers.

## MATERIALS AND METHODS

A total of 225 sexed male broiler chicks (*Cobb* strain) of a day-old age were randomly divided into 15 groups consisting of 15 chicks in each group. All the birds were provided with feed and water *ad libitum* throughout the experiment. The groups were maintained as per the following treatment schedule for 6 weeks:

Group 1: Basal diet control (1–42 days)
Group 2: FeSO_4_ (0.5% of feed) toxic control (1–42 days)
Group 3: FeSO_4_ (1–28 days) + *Withania somnifera* at 0.1% of feed (29–42 days)
Group 4: FeSO_4_ (1–28 days) + *Ocimum sanctum* at 0.1% of feed (29–42 days)
Group 5: FeSO_4_ (1–28 days) + *Asparagus racemosus* at 0.1% of feed (29–42 days)
Group 6: FeSO_4_ (1–28 days) + *Andrographis paniculata* at 0.1% of feed (29–42 days)
Group 7: FeSO_4_ (1–28 days) + *Murraya koenigii* at 0.1% of feed (29–42 days)
Group 8: FeSO_4_ (1–28 days) + shilajit at 0.1% of feed (29–42 days)
Group 9: FeSO_4_ (1–28 days) + *Gymnema sylvestre* at 0.1% of feed (29–42 days)
Group 10: FeSO_4_ (1–28 days) + *Allium sativum* at 0.1% of feed (29–42 days)
Group 11: FeSO_4_ (1–28 days) + Spirulina at 0.1% of feed (29–42 days)
Group 12: FeSO_4_ (1–28 days) + (*W. somnifera* + *As. racemosus* + *An. paniculata* at 0.05% of each in feed for 29–42 days)
Group 13: FeSO_4_ (1–28 days) + (*W. somnifera* + *M. koenigii* +*Al. sativum* at 0.05% of each in feed 29–42 days)
Group 14: FeSO_4_ (1–28 days) + (Spirulina + shilajit + *G. sylvestre* at 0.05% of each in feed for 29–42 days)
Group 15: FeSO_4_ (1–28 days) + (*An. paniculata* + *O. sanctum* + *As. racemosus* at 0.05% of each in feed 29–42 days)

The blood samples were drawn from wing vein at the end of 4^th^ and 6^th^ weeks from the birds in each group for the assay of superoxide dismutase (SOD)[6] and catalase.[7] Sera samples were separated from the blood for the estimation of alanine transaminase (ALT) and serum creatinine by using diagnostic kits (Qualigens Pvt. Ltd., Mumbai, India). The birds were sacrificed at the end of 6^th^ wk and tissues were collected for the assay of reduced glutathione (GSH)[8] and thiobarbituric acid reactive substances (TBARS)[9] in liver and kidney homogenates.

The data were subjected to statistical analysis by applying one-way analysis of variance (ANOVA) using statistical package for social sciences (SPSS), 10^th^ version. Differences between means were tested using Duncan’s multiple comparison test and significance was set at *P*<0.05.

## RESULTS AND DISCUSSION

The concentrations of TBARS [nanomoles malondialdehyde (MDA)/g protein] of liver and kidney in basal diet control (group 1) were 153.42±5.943 and 106.709±3.788, respectively, which was significantly (*P*<0.05) increased in iron toxic control group 2 (185.256±3.796 and 177.763±3.306, respectively) at the end of 6^th^ wk. Groups 3-15 that were supplemented with herbs and their combinations during the last 2 weeks following discontinuation of iron revealed a significant (*P*<0.05) decrease in the concentration (from 140.145±3.172 to 154.777±6.680 and from 119.247±5.725 to 142.039±4.62 in liver and kidney, respectively) as compared to group 2.

The concentrations of GSH (mg/g protein) in liver and kidney in the basal diet control (group 1) were 70.425±2.471 and 31.573±1.215, respectively, which was significantly (*P*<0.05) reduced in iron toxic control group 2 (33.237±1.557 and 18.707±0.59, respectively) at the end of 6^th^ wk. Groups 3–15 that were supplemented with herbs and their combinations during the last 2 wk following discontinuation of iron revealed a significant (*P*<0.05) increase (from 60.683±2.129 to 69.095±2.099 and from 24.740±0.938 to 31.274±0.668 in liver and kidney, respectively).

The activities of erythrocytic SOD (IU/g protein) and catalase (μM/min) at the end of 4^th^ wk in the basal diet control (group 1) were 41.158±0.471 and 2.55±0.055, respectively, which were significantly (*P*<0.05) increased in all the remaining groups with the values ranging from 64.187±0.345 to 66.305±0.69 and from 3.464±0.013 to 3.868±0.084, respectively. However, following supplementation with herbs and their combinations in test, there was a significant (*P*<0.05) decrease in the activity of SOD in groups 3-15 at the end of 6^th^ wk with the values ranging from 55.288±0.52 to 56.883±0.531 and from 2.852±0.045 to 3.085.±0.092, respectively, as compared to their corresponding 4^th^ wk values and the activity in iron toxic control group 2 (81.852±0.834 and 4.312±0.178, respectively).

In this study, the lipid peroxidation biomarkers like TBARS and the antioxidant defenses like SOD and catalase were elevated, while GSH was reduced in the toxic control group that was maintained on iron throughout the study. All the changes in the antioxidant defense profile were significantly reversed when treated with herbs and herbal combinations. These results are attributed to the antioxidant properties of steroidal lactones such as withanolides, which are the main active components of *W. somnifera*.[10] *O. sanctum* has been reported to reduce lipid peroxidation and increase GSH concentration in blood.[11] *An. paniculata* has been reported to increase the activities of SOD, catalase, glutathione peroxidase and glutathione reductase as well as the concentration of glutathione, with a subsequent decrease in lipid peroxidation. Similar reports were documented for shilajit in rats.[12] Spirulina decreased the MDA levels and increased the GSH levels in the goat liver homogenate *in vitro*.[13]

The activity of ALT (units/l) in basal diet control (group 1) was 18.656±0.583 at the end of 4^th^ wk, which was significantly (*P*<0.05) increased in all the remaining groups with the values ranging from 43.142±0.865 to 44.891±1.250. However, following supplementation with herbs and their combinations in test, there was a significant (*P*<0.05) decrease in the activity of ALT in groups 3-15 at the end of 6^th^ wk with the values ranging from 36.729±2.633 to 39.644±1.475 as compared to their corresponding 4^th^ wk values and the activity in iron toxic control group (66.462±1.56). Amongst all the groups, the activity remained significantly (*P*<0.05) lower in basal diet control (24.778±0.949). The activity of ALT was determined to assess the degree of damage to the liver as the levels of certain enzymes like alanine transaminase (ALT), aspartate transaminase (AST), gamma glutamyl transferase (GG T), etc., are reported to be elevated following hapatocellular injury.[14] In this study, the activity of ALT was significantly elevated in the iron toxic control group, suggesting the hapatocellular insult following administration of iron. Treatment with herbs and their combinations following discontinuation of iron resulted in significant reduction in the activity of ALT. The hepatocellular injury due to iron could be attributed to the iron-induced generation of ROS or free radicals and the reversal of the findings following treatment could be attributed to the antioxidant and the hepatoprotective potential of the herbs[15] to prevent iron-induced toxic manifestations, though there was no complete prevention of changes.

The serum creatinine concentration (mg/dl) in the basal diet control (group 1) was 0.402±0.016 at the end of 4^th^ wk, which was significantly (*P*<0.05) increased in all the remaining groups with the values ranging from 1.274±0.034 to 1.385±0.106. However, following supplementation with herbs and their combinations in test, there was a significant (*P*<0.05) decrease in the concentration of serum creatinine in groups 3–15 at the end of 6^th^ wk, with the values ranging from 1.000±0.011 to 1.137±0.009 as compared to their corresponding 4^th^ wk values and that of iron toxic control group 2 (2.154±0.075). Amongst all the groups, the concentration remained significantly (*P*<0.05) lower in basal diet control (0.573±0.038) at the end of 6^th^ wk. Lowest concentration of serum creatinine was recorded in group 12 (1.000±0.011) at the end of 6^th^ wk. In the present study, serum creatinine was significantly increased in the toxic controls at 4^th^ wk, which could be attributed to the free radical induced oxidative damage by iron on kidney. These findings can be further substantiated from the results of oxidative stress which indicated elevated TBARS and reduced GSH. The groups that were treated with herbs and herbal combinations following discontinuation of iron resulted in significant decrease in serum creatinine as compared to toxic control group, which further confirms the therapeutic potential of the herbs in test. The beneficial renal protective actions of herbs in test may be attributed to their antioxidant/free radical scavenging actions and protection of protein thiols from deleterious action of iron in kidney.

In conclusion, the study revealed that iron induces toxicity to liver and kidney by means of oxidative stress by either generating excess free radicals or by disturbing antioxidant defenses and supplementation of herbs with antioxidant potential is useful in the management of accidental iron toxicity in broilers.
